# Role of polymorphonucleates in the pathogenesis of systemic juvenile idiopathic arthritis and Still's disease: a proof of concept study

**DOI:** 10.1186/1546-0096-13-S1-P56

**Published:** 2015-09-28

**Authors:** F Magnotti, OM Lucherini, C De Clemente, R Talarico, G Emmi, M Galeazzi, R Cimaz, L Cantarini

**Affiliations:** 1 University of Siena, Department of Medical Science, Surgery and Neurosciences, Siena, Italy; 2 University of Pisa, Pisa, Italy; 3 University of Florence, Florence, Italy

## Background

Systemic juvenile idiopathic arthritis (sJIA) is an autoinflammatory disorder, characterized by neutrophilia and abnormal innate immunity response. Its counterpart in adult patients is the adult-onset Still's disease (AOSD). It has been hypothesized a pathogenic role of neutrophils in both conditions, because of patients neutrophilia, maybe related to the typical higher production of pro-inflammatory cytokines, like IL-1β, whose role in these disorders should explain the efficacy of IL-1 blockers. IL-1β is synthesized as inactive form and its activation is mediated by the NLRP3 inflammasome. Increased release of this cytokine in the extracellular environment lead to a positive feedback loop that perpetuates and amplifies itself stimulation. This mechanism as well as neutrophils’ activation state could be modified in sJIA and AOSD.

## Objectives

Our aim was to verify possible differences between sJIA and Still's patients compared to healthy donors, in term of PMNs responsiveness to the extracellular environment and activation state.

## Methods

PMNs were obtained from heparinised venous blood of sJIA patients (n=6), AOSD patients (n=8) and healthy controls (HC, n=14). All patients’ samples were collected during stages of active or non-active disease, according to international disease activity criteria used for the assessment of each disease. After lipopolysaccharide (LPS) treatment, IL-1β content in PMNs supernatants was measured by ELISA. CD11b and CD66b expression levels were measured by flow cytometry, together with intracellular ROS levels through the H_2_DCFDA ROS-indicator.

## Results

In comparison with HC, sJIA and AOSD PMNs showed an increased IL-1β secretion after LPS or LPS+ATP stimulation (p<0.05). Reduced IL-1β secretion was observed through caspase-1 inhibitor pre-treatment. About neutrophils activation state, higher intracellular ROS levels were detected in PMNs of sJIA patients (p<0.05) than HC, at basal condition. Moreover, also CD11b and CD66b surface marker expression levels, were higher at baseline in sJIA respect HC (p<0.01 and p<0.05 respectively). Instead, no differences were obtained between AOSD and HC PMNs.

## Conclusions

Data from our proof of concepts study suggest a possible involvement of PMNs in the pathogenesis of sJIA and AOSD, since they seem more sensitive to pro-inflammatory stimuli respect healthy controls. An important difference interests PMNs’ activation state, that seems higher in sJIA but comparable to HC in AOSD. Further studies are necessary to confirm and validate these results, but the trend observed may have a potential role in the direction of future therapeutic studies.

